# Novel Extruded Starch-Beet Pulp Composites for Packaging Foams

**DOI:** 10.3390/ma13071571

**Published:** 2020-03-29

**Authors:** Boussad Abbès, Catherine Lacoste, Christophe Bliard, Chadi Maalouf, Florica Simescu-Lazar, Fabien Bogard, Guillaume Polidori

**Affiliations:** 1 UFR SEN, SFR Condorcet FR CNRS 3417, Université de Reims Champagne-Ardenne, Moulin de la Housse, 51687 Reims, France; chadi.maalouf@univ-reims.fr (C.M.); florica.lazar@univ-reims.fr (F.S.-L.); fabien.bogard@univ-reims.fr (F.B.); guillaume.polidori@univ-reims.fr (G.P.); 2 ESIReims, 3 esplanade Roland Garros, 51100 Reims, France; catherine.lacoste@univ-reims.fr; 3 ICMR-UMR 7312 CNRS, SFR Condorcet FR CNRS 3417, Université de Reims Champagne-Ardenne, Moulin de la Housse, 51687 Reims, France; christophe.bliard@univ-reims.fr

**Keywords:** starch, beet pulp, extrusion, biobased composite

## Abstract

This article concerns the elaboration and the characterization of a novel biobased potato starch-beet pulp composite for packaging applications as cushion foams. A twin-screw extruder was used to elaborate composite foams. SEM observations of these materials were conducted, and thermomechanical properties were studied in terms of thermal transitions (TGA, DSC) and viscoelastic properties (DMA). The effect of relative humidity content on viscoelastic properties was analyzed as a function of frequency. The different test results show that the composite structures are homogeneously mixed. The sponge-like structure of the beet-pulp disappears indicating a good compatibility between the two mixed constituents. The DSC curve of starch-beet pulp foam shows a single thermal transition at 153.6 °C, indicating the thermal homogeneity of the obtained composite material. The density value of starch-beet pulp foam is higher than conventional foams, but this can be optimized by adjusting the technological parameters of the extruder. The viscoelastic properties of the developed materials depend on the relative humidity.

## 1. Introduction

The packaging industry is looking for materials with specific properties (strength, lightness, impact protection, etc.) that can be easily disposed of after use while being environmentally friendly. At the same time, consumers have developed an environmental conscience and have now become aware of the need to use biodegradable packaging [1]. Packaging has thus become a societal issue. Research teams embraced this trend and numerous studies related to biopolymers have been conducted [2] to reduce the carbon footprint and pollution risks caused by using conventional polymers [3,4,5,6]. The NF-EN-13432 standard defines requirements for qualifying packaging as "biodegradable and recoverable in industrial composting" [7,8]. The most suitable objects that can be replaced by biodegradable materials are single-use packaging materials [9,10]. Today, the materials are mainly made of expanded polystyrene with a low density and interesting impact resistance and thermal insulation characteristics [2]. Significant efforts have been made in recent years to develop environmentally friendly starch-based foams to replace polystyrene foams, due to the large amount of polystyrene used in foam packaging as well as its difficulty in collecting and to recycling [11]. Starch is obtained from different sources (potato, rice, manioc, wheat, corn, etc.) [12], and different techniques such as twin-screw extrusion [13,14], compression/explosion processing [15], microwave heating process [16], freeze-drying [17], and supercritical fluid extrusion [18] are used to develop starch foams with different properties and cellular structures. Foams obtained with potato and cassava starches have lower densities than those obtained from cereals such as corn [19]. To improve the mechanical properties of the starch-based foams, various natural fibers such as flax, soft wood, jute, aspen, etc., have been incorporated in starch-based biocomposite systems [11]. Furthermore, sugar beet pulp was successfully used as filler phase with different matrix phases to produce biocomposites [20,21,22]. In fact, sugar manufacturing process, derived from sugar beet, generates large quantities of solid pulp by-product at the end of the sugar extraction process. Dried beet pulp pellets are mainly used for animal feed or biogas generation [23,24,25]. It is then important to find new alternatives to profitably use the large amounts of this low-value byproduct.

**Figure 1:**
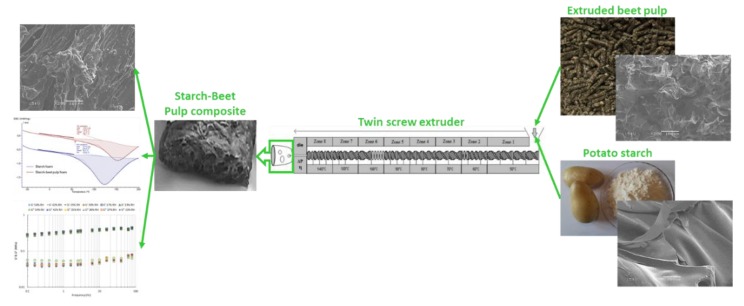
Schematic representation of the overall work.

The main objective of this exploratory study is the elaboration and the characterization of a novel foamed biobased composite made of beet pulp and potato starch used as cushioning for industrial goods (Figure 1). In this composite, beet pulp provides mechanical properties to the composite, and starch acts as a binder to stick the fibers together and assure the transfer of shear forces between them. This cushioning material is composed of renewable materials, fully biodegradable with planned obsolescence.

In this study, starch-beet pulp composite foams were prepared by extrusion. The composites were characterized for physical, structural, and mechanical properties.

## 2. Materials and Methods

### 2.1. Materials

#### 2.1.1. Extruded Beet Pulp

**Figure 2:**
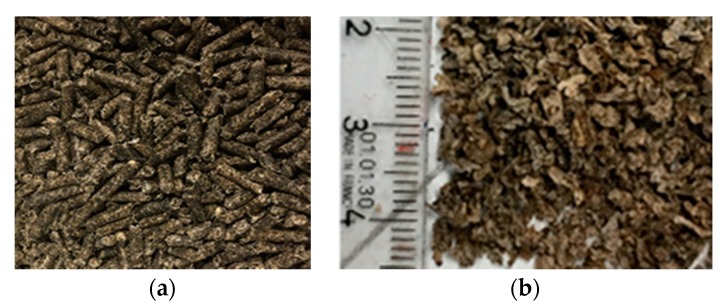
(**a**) Extruded beet pulp pellets; (**b**) Extruded beet pulp pellets after drying.

The extruded beet pulp (BP) pellets were purchased from Cristal Union factory (Pomacle, France) with 8–10 mm diameter and 18% humidity (Figure 2) during the 2017–2018 sugar beet campaign. The extruded pulp is mainly composed of pectin, cellulose and hemicellulose [26]. To ensure proper conservation, the pellets were kept in the laboratory at −20 °C until use. The extruded BP were immersed in 3.5 times their weight of water for two hours to ensure a complete swelling and then dried at 50 °C for two days in a pulsed air tower [20] before use.

#### 2.1.2. Potato Starch

Potato starch (15% humidity) was provided by Roquette (Lestrem, France). This starch was chosen among other botanical sources because of its high polymerization degree and high viscous properties providing interesting mechanical properties for the Starch-Beet Pulp (SBP) composite. In SBP composite starch acts as a binder to stick the particles together and assure the transfer of shear forces between the fibers.

#### 2.1.3. Composite Formulation

Potato starch and potato SBP composite foams were prepared as follows: to 10 parts of dried swollen pulp obtained from extruded beet pulp was added 3 parts of water and 10 parts of a 7/5 starch-glycerol mix previously dried for 2 h at 120 °C in an oven. Glycerol with a density of 1.26 g/cm^3^, provided by A.D society (France), was used as a plasticizer. The obtained admixture was mechanically stirred to homogenize the distribution of the different constituents in the SBP blend.

### 2.2. Methods

#### 2.2.1. Extrusion Setup

**Figure 3:**
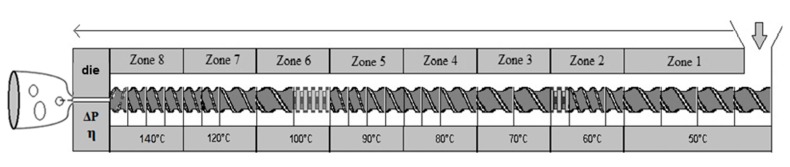
Configuration of the co-rotating twin-screw extruder machine.

**Figure 4:**
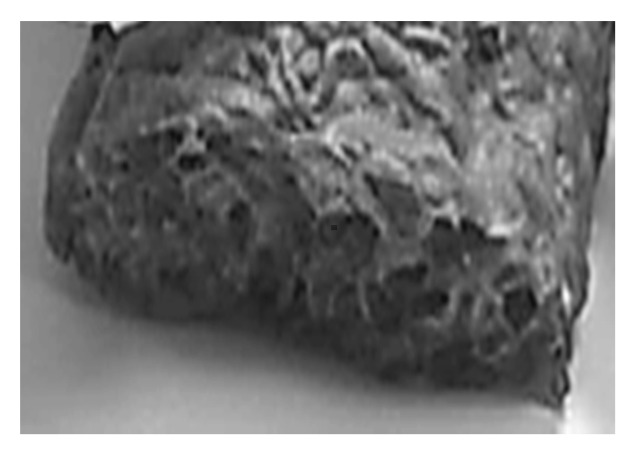
Photograph of the SBP composite foam.

The SBP mix were compounded using a Clextral BC21 co-rotating twin-screw extruder machine. The configuration of a mixing and suited transport screw elements is shown in Figure 3. The barrel temperature was set between 70 °C to 120 °C, and the compound was extruded through a ribbon die geometer. After extrusion, the ribbons were cut into 15 cm length and 2 cm width wise strips, identified and conditioned for one week at 23 ± 2 °C and 50 ± 10% relative humidity according to NF EN ISO 7214 norm before characterization. A photograph of the extruded SBP composite foam is shown in Figure 4.

#### 2.2.2. Foam Density

Bulk density, also called apparent density or volumetric density, is defined as the mass of the material divided by the total volume it occupies. It was calculated as the mass of an extruded SBP (ESBP) ribbon divided by its geometric external measured volume. Absolute density is obtained when the volume measured excludes the pores as well as the void spaces between material within the bulk sample. In this study, the absolute density was determined using the Bourdot et al. pycnometer method [27] particularly well adapted for structurally complex multi-hollow fibrous biological materials. In this method, the pycnometer flask is filled with a given mass of dried beet-pulp (approximately 20 g) and 50% of its volume with cyclohexane. Cyclohexane being a non-polar solvent does not affect the composition and he mass of the pulp. This mixture is subjected to six boiling (30 min) and cooling (10 min) cycles. During the cycles, air escapes from the beet-pulp cells allowing cyclohexane to occupy the pore spaces. In the sixth cycle, the system is kept under an argon atmosphere to avoid the reentry of humidity. At the end of the cycles, the pycnometer is filled up with cyclohexane at room temperature (20 °C) and plugged with the stopper. The system (approximately 150 g) is then weighed using a balance with an accuracy of 10^−3^ g. The absolute density is calculated using the following equation:

(1) $$\rho_{abs}=\frac{m_{1}\times \rho_{cyc}}{m_{1}-\left(m_{2}-m_{3}\right)}$$

where $\rho_{abs}$ is the absolute density (kg·m^−3^), $\rho_{cyc}$ is the density of cyclohexane (kg·m^−3^), $m_{1}$ is the dry mass of SBP aggregates, $m_{2}$ is the mass of the pycnometer filled with cyclohexane and saturated aggregates, and $m_{3}$ is the mass of pycnometer and cyclohexane.

#### 2.2.3. Microscopy Observations

Scanning electron microscopy (SEM) observations were carried out on a 15kV JEOL/JSM 6460LA (JEOL (Europe) SAS, Croissy, France). All samples were dried at 60 °C for 48 h in a vacuum desiccator, then coated with carbon to increase their electrical conductivity.

#### 2.2.4. Thermogravimetric Analysis Procedure

TGA experiments were conducted using a TGA (NETZSCH-TG 209 F3 Tarsus) (NETZSCH-Gerätebau GmbH, Paris, France) to evaluate the moisture content and the thermal stability of potato starch and starch-beet pulp composite foams. The measurements were carried out in triplicate on 10–15 mg samples put in an aluminum pan and heated from 25 °C to 600 °C with a heating rate of 10 °C/min in a nitrogen atmosphere.

#### 2.2.5. Differential Scanning Calorimetry Procedure

The thermal characteristics of potato starch and starch-beet pulp composite foams were determined by using a DSC (NETZSCH – DSC 204 F1 Phoenix) (NETZSCH-Gerätebau GmbH, Paris, France), after 48 h conditioning at a temperature of 23 °C and 65% relative humidity. Aluminum sealed pans were used to test in triplicate samples of 5–40 mg. The heat flow was measured as function of the temperature and time. The samples were heated from −80 °C to 200 °C at a heating rate of 20 °C/min. A reference empty DSC pan was used to balance the heat capacity of the sample pan. Proteus V4.8.5 NETZSCH software is used to analyze the results.

#### 2.2.6. Dynamic Mechanical Analysis Procedure

DMA tests were carried out in a DMA Q800 (TA Instruments, Guyancourt, France), with DMA-RH accessory for humidity and temperature control. This makes it possible to determine the mechanical properties of the samples under controlled humidity and temperature conditions.

To compare the viscoelastic properties of the different foams, creep/recovery tests were performed using the compression clamp kit which is suitable for low to moderate modulus materials such as foams. In this mode, the sample is placed on a fixed flat surface of 40 mm diameter and an oscillating plate applies a defined force. The samples were cut into 1.22 cm^2^ circular slices of 5 mm thickness. The samples were preloaded with a force of 0.01 N and then assigned a constant stress of 10 kPa for 5 min at a constant temperature of 23 ± 2 °C and variable relative humidity conditions ranging from 30% to 52% RH. After 5 min, the stress was removed, and the samples undergo 15 min recovery. The creep compliance of the tested materials was measured.

To assess the viscoelastic properties as a function of frequency, we have also performed tests in the multi-frequency mode (0.1 Hz–100 Hz), with a constant oscillation stress amplitude of 10 kPa at a constant temperature of 23 ± 2 °C and variable relative humidity conditions ranging from 13% to 54% RH. The storage and loss moduli of the materials were measured.

## 3. Results and Discussion

### 3.1. Microscopic Structure of the Extruded Foams

The following SEM images make it possible to visualize the microscopic structure of the raw materials and their physical transformation after the extrusion process.

**Figure 5:**
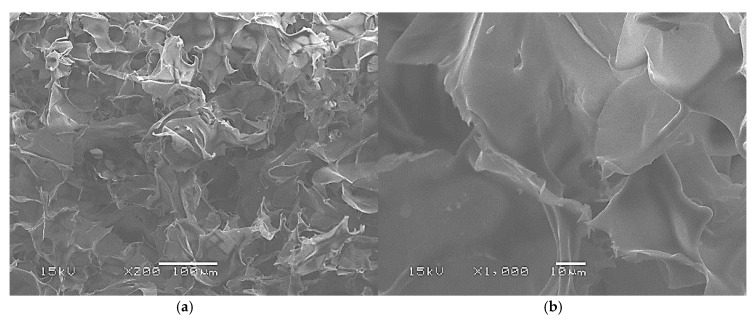
Dry lyophilized starch raw material SEM micrographs: (**a**) ×200; (**b**) ×1000.

**Figure 6:**
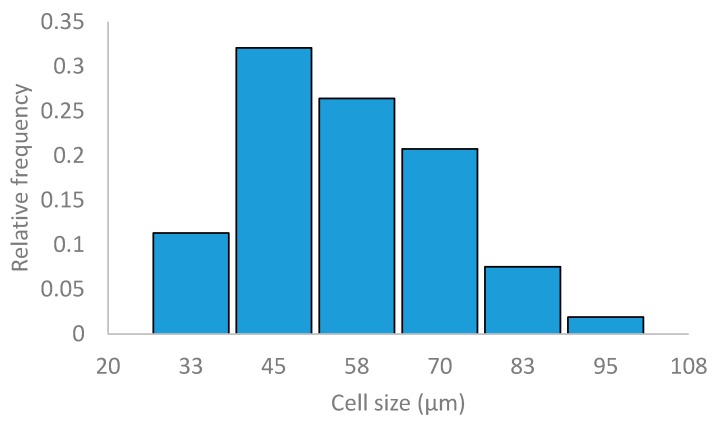
Cell size frequency distribution of dry lyophilized starch raw material.

The dry lyophilized starch raw material SEM micrographs are shown in Figure 5 with two different magnifications. These SEM micrographs are characteristic of a foamed material with a homogeneous distribution of the cells. The cell size frequency distribution of this material obtained by using ImageJ software (version 1.52a) is shown in Figure 6 with an average cell size of 62 ± 14 µm.

**Figure 7:**
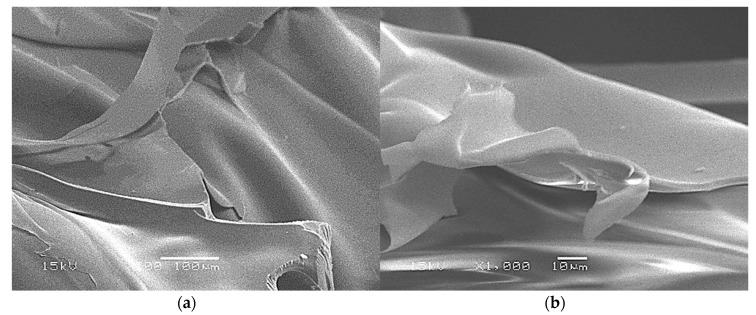
Plasticized starch SEM micrographs: (**a**) ×200; (**b**) ×1000.

After plasticization of starch, the cell structure observed in Figure 5 disappears, leaving a corrugated sheet structure as shown in Figure 7. This corrugated sheet structure is characteristic of a plasticization obtained by the mechanical action of the extrusion screw in the presence of water and glycerol [13,14].

**Figure 8:**
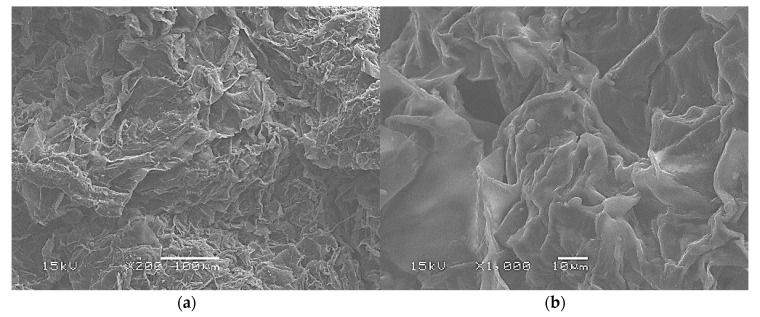
Beet pulp raw material SEM micrographs: (**a**) ×200; (**b**) ×1000.

**Figure 9:**
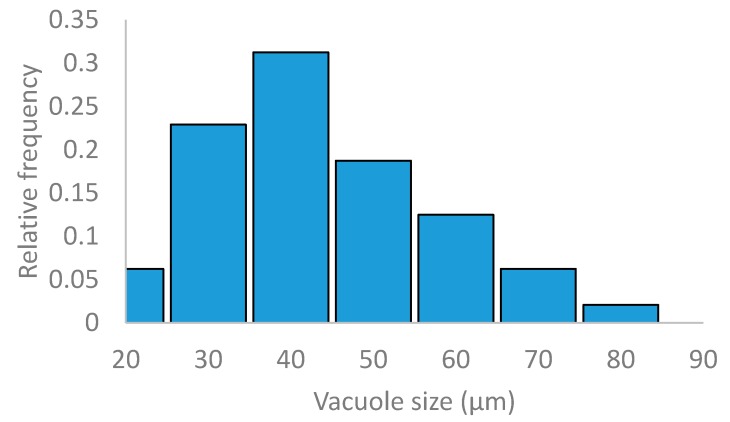
Vacuole size frequency distribution of beet pulp raw material.

Beet pulp has a homogeneous sponge-like native structure as shown by the SEM micrographs given in Figure 8. This structure results from the destructuring undergone by the beet during the industrial sugar extraction process. The typical cellulose rectilinear fibrous structure is not observed in the micrographs. The vacuole size frequency distribution of the beet pulp is plotted in Figure 9 giving an average cell size of 49.6 ± 13.5 µm.

**Figure 10:**
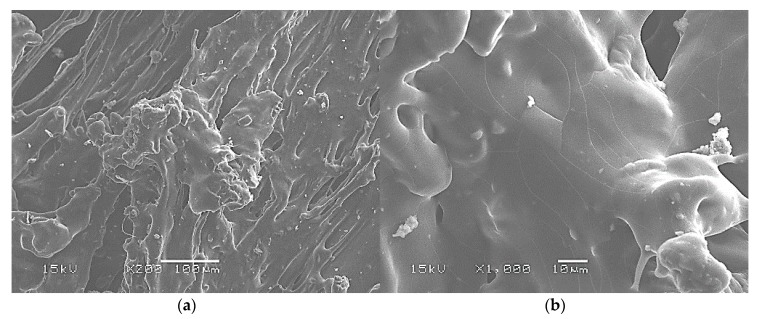
Potato starch-beet pulp composite foam SEM micrographs: (**a**) ×200; (**b**) ×1000.

The potato starch-beet pulp composite foam structure is given in Figure 10. The previous structures are homogeneously mixed, and the sponge-like structure of the beet pulp disappear indicating the good miscibility between the two mixed constituents.

### 3.2. Density Analysis

Table 1 gives the bulk and absolute density values for extruded starch beet pulp foam (ESBP) compared to the ones of native starch, and beet pulp [20]. The absolute density of the composite (starch + beet pulp) is lower than that of raw starch, because the absolute density of beet pulp is lower than that of raw starch. The bulk density of the composite is higher than that of raw starch because the expansion of the extruded composite is not enough and needs to be improved. The density value of starch-beet pulp foam is still higher than conventional foams. This poor value is the result of preliminary tests on the feasibility of extrusion with no special focus on the foam characteristics. The density of the obtained foam could be largely optimized by adjusting the technological parameters at the exit of the extrusion die since the mixture tended to collapse. A physical or a chemical agent such as an expanding gas (CO_2_ for example) can also be added in the extrusion process to improve the foam expansion and decrease the density value.

### 3.3. Thermogravimetric Analysis Results

**Figure 11:**
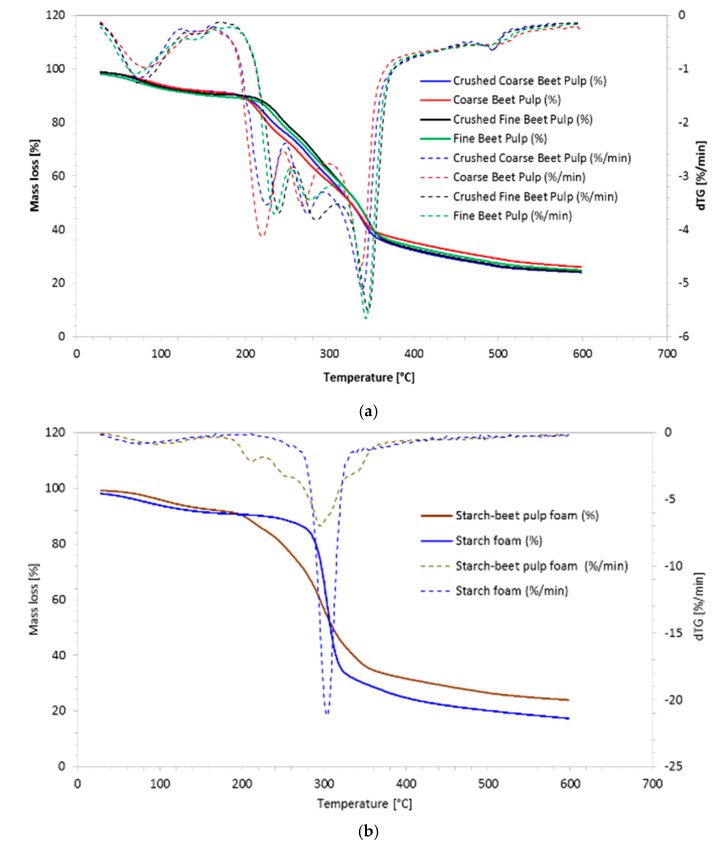
TGA thermograms: (**a**) beet pulps; (**b**) starch and starch-beet pulp foams.

TGA thermograms of beet pulps are given in Figure 11a showing three consecutive thermal zones corresponding to water loss (100–200 °C), followed by the substrate decomposition (200–350 °C) and finally the material carbonization (>350 °C). The water loss of the beet pulp is about 10% at 100 °C. The beet pulp undergoes several decompositions between 200 °C and 350 °C due to the fragility of its constituents, such as the pectin and hemicellulosic fractions. Similar results were obtained by Sidi-Yacoub et al. [28]. The native beet pulp loses mass earlier because it has not undergone any preliminary degradation by mechanical energy. This is clearly shown by the temperature shift of about 20 °C observed between the mass loss curves and confirmed by derivative peaks shift (dotted lines).

Figure 11b shows a typical TGA curve obtained for starch foam with slow water loss between 100 °C and 200 °C, followed by sudden decomposition at 300 °C and finally the material carbonization (>300 °C). These thermograms are typical for potato starch as it can be found in the work of Liu et al. [29]. The TGA curve obtained for the composite starch-beet pulp foam corresponds to the superposition of the beet pulp and starch foam thermograms without mutual influence between the two materials.

### 3.4. Differential Scanning Calorimetry (DSC) Results

**Figure 12:**
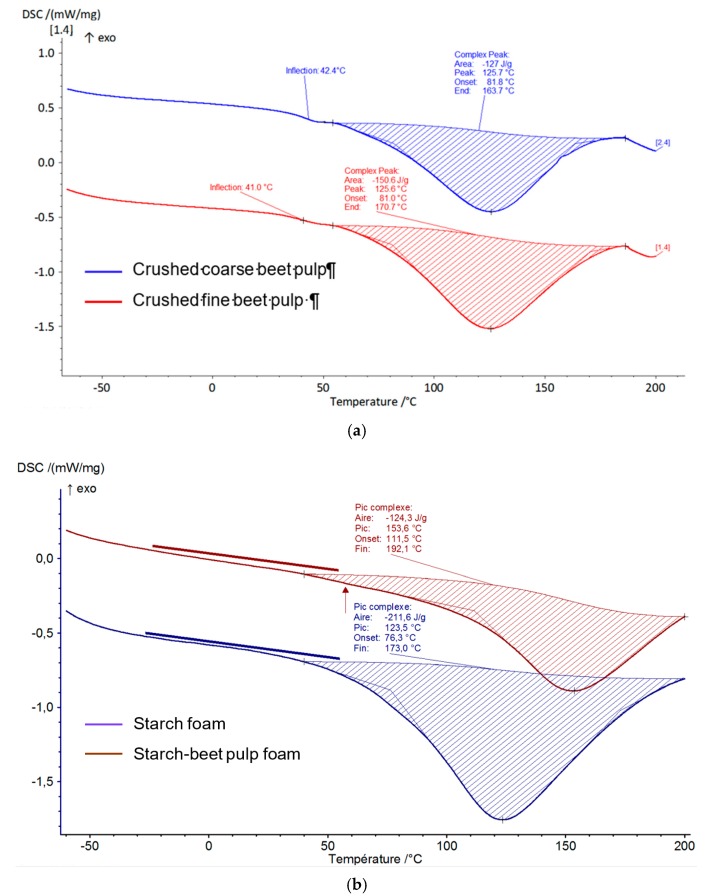
DSC thermograms: (**a**) beet pulp; (**b**) starch and starch-beet pulp foams.

DSC is one of the most used techniques for the analysis of thermal transition of polymers and biopolymers such as starch-based and beet pulp-based materials [30,31]. Typical DSC curves of beet pulp, starch and the composite resulting from their mixture are given in Figure 12. The peak temperatures corresponding to the thermal transition of beet pulp and starch are approximately the same: 123.5 °C for starch and 125.6 °C for beet pulp. The DSC curve of starch-beet pulp foam in Figure 12b shows a single thermal transition at 153.6 °C, indicating the thermal homogeneity of the obtained composite material.

### 3.5. Dynamic Mechanical Analysis (DMA) Results

#### 3.5.1. Storage and Loss Moduli

**Figure 13:**
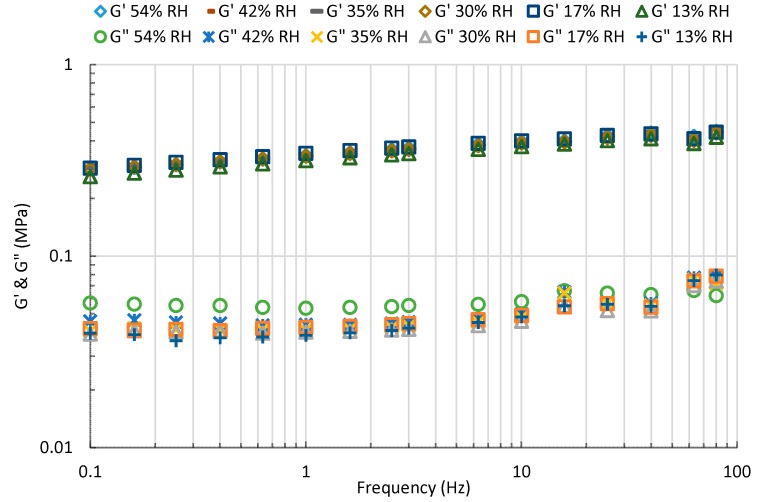
Storage modulus (G’) and Loss modulus (G”) of starch-beet pulp foams at different RH conditions.

**Figure 14:**
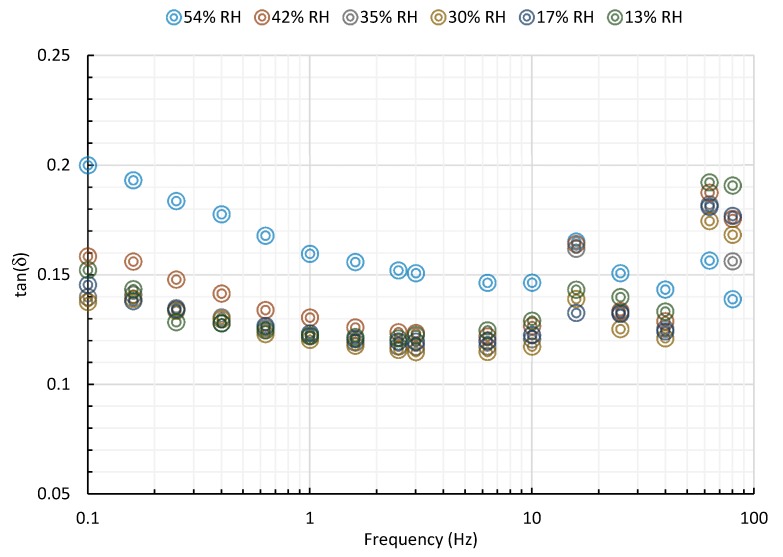
tan(δ) of starch-beet pulp foams at different RH conditions.

The storage modulus represents the energy stored in the elastic structure of the sample. When it is higher than the loss modulus the material can be considered as mainly elastic. The loss modulus is the viscous part and represents the amount of energy dissipated in the sample. The storage (G’) and loss (G”) moduli of starch-beet pulp foams at different RH conditions are given in Figure 13. It can be seen that the effect of relative humidity of the composite material on the viscoelastic properties is small. The ratio of the moduli (G”/G’) is defined as tan(δ), and indicates damping or the relative degree of energy dissipation of the material. As shown in Figure 14, this ratio varies between 0.1 and 0.2 in the range of the tested RH.

#### 3.5.2. Creep-Recovery Response

**Figure 15:**
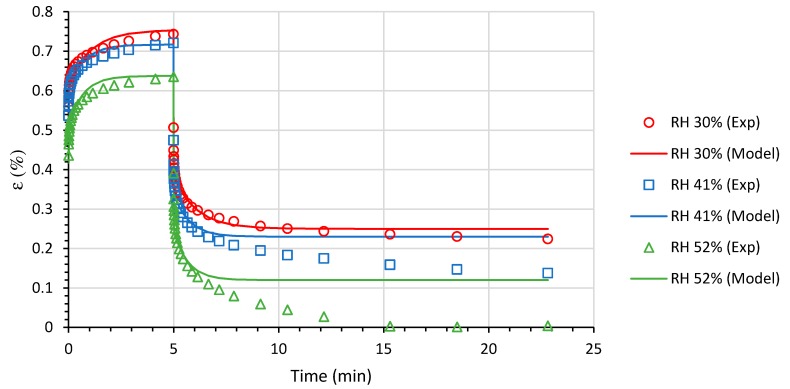
Creep-recovery curves of starch-beet pulp foams at different RH conditions.

The creep-recovery curves of starch-beet pulp composite foams at different RH conditions are plotted in Figure 15. When the relative humidity increases, the composite foam show lower compliance allowing less deformation under stress. In investigations carried out by Mitrus [32] it was found that blend moisture was observed to influence the maximum stress generated in a compressed thermoplastic starch. The stresses produced with increasing mixture moisture are higher, but a faster decrease in stress values with increasing glycerol content was reported. The results showed that an increase in glycerol content is accompanied by a lower tensile strength of the thermoplastic starch. Although an increase in water content may improve the strength of the produced material, it is even more likely that some boundary value of total plasticizer content exists and that when it is exceeded the material will become softer.

The creep-recovery response of the foams can be modelled using the viscoelastic generalized Kelvin-Voigt model given by the following equations for creep ($\varepsilon_{c}\left(t\right)$) and recovery ($\varepsilon_{r}\left(t\right)$), respectively:

(2) εc(t)=σ0(J0+tη0+∑i=1nJi(1−e−tθi))

(3) εr(t)=σ0(J0−∑i=1nJie−tθi(1−eτθi)),  t>τ

where t is the time, $\sigma_{0}$ is the creep stress, $\eta_{0}$ is the viscosity, $J_{i}$ are the compliances, $\theta_{i}$ are the retardation times, and τ is the creep loading time.

The obtained results are summarized in Table 2. Creep and creep recovery are irreversible, giving a permanent deformation of the sample. Extent of deformation recovery is governed by the strength of the physical network in the starch-beet pulp composite foams. In fact, it can be seen that the ratio of non-recovered strain at the end of experiment $\left(\varepsilon_{nr}\right)$ to the total strain before removing the load $\left(\varepsilon_{c}\right)$ decreases with increasing RH. This accounts for the irreversible part of the deformation which is higher for 30% RH and tends to vanish for 52% RH.

## 4. Conclusions

We proposed in this study a composite foam material extruded from beet-pulp and starch as an alternative to polymer foams such as expanded polystyrene (EPS), loose-fill (foamed chips for filling space around goods within a packing box) used in cushion packaging. The extrusion process of the beet-pulp/plasticized starch compound produces a homogeneous foamy material. These results were obtained with a non–optimized process and can be improved by adjusting the technological parameters at the exit of the extrusion die since the mixture tended to collapse after extrusion. A physical or a chemical agent such as an expanding gas (CO_2_ for example) can also be added in the extrusion process to improve the foam expansion and decrease the value of the density.

## Figures and Tables

**Table 1:** Material densities.

ESBP Foam Bulk Density (g/cm^3^)	Raw Starch Bulk Density (g/cm^3^)	BP Bulk Density [20] (g/cm^3^)
0.686 ± 0.05	0.510 ± 0.05	0.194 ± 0.007
SBP foam abs. density (g/cm^3^)	Raw Starch abs. density (g/cm^3^)	BP abs. density [20] (g/cm^3^)
1.470 ± 0.005	1.510 ± 0.005	1.073 ± 0.005

**Table 2:** Generalized Kelvin-Voigt model parameters.

RH (%)	εnrεc	$J_{0}\;({\mathrm{MPa}}^{-1})$	$\eta_{0}\;(\mathrm{MPa}.\min)$	$J_{1}\;({\mathrm{MPa}}^{-1})$	$\theta_{1}\;(\min)$	$J_{2}\;({\mathrm{MPa}}^{-1})$	$\theta_{2}\;(\min)$
30	0.50	2499.4	1.04	3671.6	0.00076	1366.9	1.085
41	0.29	2299.0	1.05	3751.6	0.00075	1115.4	0.732
52	0.01	1200.0	1.07	3881.4	0.00075	1291.0	0.674
